# A literature review of the research on students’ evaluation of teaching in higher education

**DOI:** 10.3389/fpsyg.2022.1004487

**Published:** 2022-09-29

**Authors:** Luying Zhao, Pei Xu, Yusi Chen, Shuangsheng Yan

**Affiliations:** ^1^Marxism School, China Pharmaceutical University, Nanjing, China; ^2^School of Health Economics and Management, Nanjing University of Chinese Medicine, Nanjing, China; ^3^School of Science, China Pharmaceutical University, Nanjing, China

**Keywords:** students’ evaluation of teaching, review, comments, higher education, indicator systems

## Abstract

Students’ evaluation of teaching is a teaching quality evaluation method and teacher performance evaluation tool commonly used in Chinese and foreign universities, and it is also a controversial hot issue in the field of teaching evaluation. At present, the research results of students’ evaluation of teaching in higher education are relatively rich, mainly focusing on reliability, validity and its influencing factors, construction of index system, problems in practical application and improvement strategies. The purpose of this article is to study the relevant research results of the current Chinese and foreign academic circles, in order to provide useful inspiration for the construction of the index system and practical application of the ideological and political theory course evaluation and teaching of Chinese college students.

## Introduction

Students’ evaluations of teaching (SET) is an activity for students to evaluate teachers’ teaching effect and teaching quality, including the reliability, validity, content, form, organization, and management of teaching evaluation. In the 1920s, the earliest college student evaluation system in the world began in the United States. In 1915, Purdue University in the United States gave birth to the first student evaluation scale, and in 1927 began to use the standardized student evaluation scale to evaluate teachers’ teaching, which is considered to be the beginning of the student evaluation system (De Neve, 1991; Theall et al., 2001). After the 1980s, the college student evaluation system began to be introduced into China while it was widely used in famous universities in western countries and became an important part of the western education system (Tu et al., 2019). In 2001, the Ministry of education of the people’s Republic of China issued several opinions on Strengthening Undergraduate Teaching in Colleges and universities and improving teaching quality, which clearly pointed out that students should be involved in teaching management. Many colleges and universities across the country responded positively and gradually applied student evaluation to teaching management. Relevant research was gradually enriched, and many suggestions on student evaluation were gradually adopted and implemented by colleges and universities (Wei and Liu, 2013). This article will systematically analyze the relevant theoretical achievements of the current Chinese and foreign academic circles, especially the European and American World College Students’ evaluation of teaching, in order to provide useful enlightenment for the construction of the index system and practical application of the evaluation of Ideological and political theory courses for Chinese college students.

## Research methodology: Literature analysis and logical analysis

This article is a literature review, so two main approaches have been adopted: documentary analysis and logical analysis. In terms of literature analysis, a large amount of literature has been consulted in writing this article, and as there is a large body of literature relating to Students’ evaluation of teaching in higher education, the authors has followed three principles in selecting literature to read. The first is to look at the time of publication of the literature, with priority given to those published recently; the second is to look at journals and authors, with priority given to well-known journals and authors; the third is to look at citation rates, with priority given to those with high citation rates; and the fourth is to pay particular attention to the two types of articles that hold pro and con views on SET. In terms of logical analysis, this article argues that the three most critical factors associated with SET are the reliability and validity of SET, the indicator system of SET, the problems that arise in the application of SET, and the countermeasures taken. This article argues that, as a review, the four most critical factors related to SET are reliability and validity, indicator systems, problems arising in application and countermeasures to be taken, and evaluation of the above perspectives. Therefore, the logical framework of this article is: an analysis of the reliability and validity of relevant SETs in the existing literature, an analysis of the indicator system, and an analysis of the problems associated with their practical application, an evaluation of the above-mentioned views on relevant SETs, and finally, a conclusion and recommendations.

## Research on the reliability and validity of students’ evaluation of teaching in higher education

In students’ evaluation of teaching, reliability refers to the degree to which students’ evaluation of teaching can stably reflect teachers’ actual teaching level, which is manifested in the stability or consistency of the evaluation results; Validity refers to whether students’ evaluation of teaching can achieve the expected goals and effects (Hong, 2010). Whether, it is reliable and effective is directly related to whether students’ teaching evaluation can be applied to teachers’ teaching evaluation. According to the current research results of the academic circles, although there are many doubts about the reliability of College Students’ teaching evaluation, the traditional view that its reliability is high has not been overturned. The validity is also controversial (Uttl, 2021). The mainstream view is that it is effective on the whole and has been supported by abundant literature.

### Views on the reliability of student’s evaluation of teaching in higher education

#### A skeptical view of the reliability of student’s evaluation of teaching

In the early research, due to the limitation of research design and method, scholars mostly used the average score of the class to measure the reliability of students’ teaching evaluation. Since this method ignored the differences between individual students, it exaggerated the students’ evaluation to a certain extent. Religious reliability (Hocevar, 1991). With the development and application of statistics and data analysis methods, scholars began to use more scientific measurement tools to conduct empirical research on students’ teaching evaluation reliability. Cheng and Zhang (2016) tested the reliability of the samples from three levels: “inter-student reliability,” “intra-course consistency” and “inter-item reliability,” and concluded that the reliability index inflated due to scoring inertia It cannot explain the reliability of the teaching evaluation results, but shows that the reliability measurement contains more interference information. Gao et al. (2010) and other scholars used the intraclass correlation coefficient to comprehensively evaluate the reliability of students’ teaching evaluation and found that: in various indicators, students’ scores on teachers’ teaching are relatively consistent, so they can be It is judged that its rater reliability is high.

#### A favorable view of the reliability of student’s evaluation of teaching in higher education

In contrast to Gao et al. (2010) and Morley (2012) and other scholars used the intra class correlation coefficient to comprehensively evaluate the reliability of students’ teaching evaluation and found that students’ scores of teachers’ teaching are relatively consistent in all indicators, so it can be judged that their raters have high reliability also believe that the SET tool used in universities is reasonable, reliable, and effective.

### Viewpoints on the validity of student’s evaluation of teaching in higher education

#### View of sufficient effectiveness

He (2017) pointed out in his research that college students, as classroom participants and stakeholders, have the most say in the teaching effect, and have the necessary cognitive and judgment skills, so students’ evaluation of teaching is scientific, objective and accurate. Li et al. (2017) believe that compared with other teaching evaluation models, student evaluation of teaching has a more direct and economical advantage, and establishes a teaching system that is mainly based on student evaluation and supplemented by expert evaluation and peer evaluation. A quality assurance system is available. Foreign studies have also pointed out that, from the long-term practice of the student evaluation system in many colleges and universities, although there are doubts, its effectiveness is worthy of recognition (Chau, 1997). Numerous colleges and universities in North America, Europe, and Asia are using student evaluations as a valid indicator to measure teaching effectiveness, or as one of the determinants of teacher promotion, tenure, pay-for-performance, or professional development (Chen and Hoshower, 2003). Cashin and Downey (1992) even argue that student evaluations are more reliable and valid than any other data and can be used to improve teachers’ teaching.

#### View of insufficient effectiveness

Wang and Guan (2017) and Zhou and Qin (2018) believe that students’ teaching evaluation is students’ subjective value judgment of teachers’ teaching. Students may have unclear cognition of teaching evaluation or negative random evaluation, which leads to the deviation of teaching evaluation results and is difficult to truly reflect the problems in teaching practice. Gu et al. (2021) believes that students are still in the process of knowledge accumulation, and the dislocation of teaching evaluation subjects and perspectives caused by students’ teaching evaluation makes it difficult for them to accurately grasp the information of teaching activities, resulting in evaluation distortion. Morley (2012), Spooren et al. (2013) and other foreign scholars pointed out that although the methods of measuring the effectiveness of students’ teaching evaluation in some typical studies are widely spread, some of them have logical problems, and educators have only reached a consensus on some characteristics of proving the effectiveness of teaching. Based on these characteristics, the effectiveness of students’ teaching evaluation cannot be clearly defined. Galbraith et al. (2012) also believes that the existing evidence is insufficient to support the effectiveness of SET as a general indicator to evaluate the teaching effect or student learning effect. This paragraph should be deleted) Wolfgang Stroebe (2020) also thinks that the existing evidence shows that students’ evaluation of teaching (sets) can not measure the teaching effect.

### Factors affecting the reliability and validity of student’s evaluation of teaching in higher education

Since reliability is a necessary condition for validity, the effectiveness of student evaluation of teaching needs to be supported by reliable evaluation results, so the academic circles generally consider the factors affecting reliability and validity comprehensively. According to the current research results, the influencing factors can be divided into two categories: teaching factors and non-teaching factors. Teaching factors include teaching methods, teaching contents, teaching attitudes, teaching means, etc. Non-teaching factors include students’ individual factors, such as grade, gender, specialty, academic achievement, teaching evaluation attitude, etc.; teachers’ personal factors, such as teachers’ age, gender, professional title, teachers’ favorite degree by students (Dennis, 2022); and curriculum factors, such as course form, course time, course importance, course difficulty, etc. As the teaching factors themselves belong to the content covered by the students’ evaluation of teaching, their influence on the evaluation results is positive. Therefore, the discussion of the influencing factors in the academic circles mainly focuses on the non-teaching factors that cause the deviation of the evaluation results.

Chinese scholars’ research on the influencing factors of College Students’ teaching evaluation is mainly to collect the data of influencing factor assumptions from students through questionnaires and interviews, and combined with the teaching evaluation results of specific colleges and universities, use statistical methods to select appropriate models for data analysis, so as to draw conclusions. Pan and Zhang (2016) concluded through empirical research that students’ subjective cognitive factors have a greater impact on the effectiveness of teaching evaluation than objective factors such as grade, gender, academic achievement, and so on. Li and Meng (2020) used the research method of grounded theory to draw a conclusion that students’ evaluation of teaching is affected by four factors: students, teachers, schools, and courses. If it is not handled properly, it is prone to adverse selection, which affects the effectiveness of teaching evaluation and the quality of school teaching. Long (2019) pointed out after analyzing the teaching evaluation data of students in Shantou University business school that there is no inevitable positive correlation between the teaching evaluation scores obtained by teachers and students’ grades of the course, and the teaching workload of teachers has a significant negative impact on the teaching evaluation scores.

Western scholars’ research on the influencing factors of students’ teaching evaluation is more comprehensive, systematic, and in-depth than domestic. However, because the influencing factors of students’ teaching evaluation are too numerous, and there are certain differences in the survey objects selected by different students, there is no agreement on the degree of influence of each factor. Gallagher (2000), Ginexi (2003), Heckert et al. (2006), and other scholars found through research that students’ characteristics (such as gender, personality, expected score of curriculum, emotion toward teachers, grade, learning expectation, major, attitude toward curriculum and teaching evaluation, the proportion of students participating in teaching evaluation in the total number of students, confidence in the effectiveness and influence of their teaching evaluation results, etc.), Teachers’ characteristics (such as gender, age, educational background, rank, relationship with students, charm and image, etc.) and curriculum characteristics (such as curriculum time, class size, curriculum nature, assessment form, etc.), and even whether the evaluation of teaching is anonymous, and whether the evaluated teachers participate in the evaluation process may have varying degrees of impact on the evaluation behavior of college students in a specific way (Kekale, 2000).

## Research on the indicators of student’s evaluation of teaching in higher education

In the process of college students’ teaching evaluation, reasonable teaching evaluation indicators are particularly important. It plays a key role in the accuracy and influence of teaching evaluation results, and is the premise and basis for students’ teaching evaluation to help improve teaching quality. The research and analysis of college students’ teaching evaluation index includes not only the construction of the specific content of the teaching evaluation index, but also the discussion of the theoretical principles that should be followed in the construction of the index. As the central link of college students’ teaching evaluation, the research on teaching evaluation indicators will provide valuable reference for improving students’ teaching evaluation system.

### Principles of constructing teaching evaluation indicators for college students

According to the current research results, the most common view in the academic circle on the construction principle of teaching evaluation indicators is to adhere to the “student-centered.” The view of “student-centered” originated from the “child-centered theory” of American educator and psychologist John Dewey, which emphasizes that the essential purpose of education is to promote the comprehensive and harmonious development of students (Ye, 2000). The student-centered evaluation index system requires that the evaluation scale should be designed from the perspective of students, based on students’ cognitive level and actual needs, and based on students’ real feelings and gains. Students’ development should become the starting point and foothold of building the teaching evaluation index system (Lv, 2014).

In addition to the mainstream views, scholars such as Wu et al. (2015) also believe that the design of the index system of college students’ teaching evaluation should at least include the characteristics of orientation, academic, interaction, difference, measurability and growth. Jiang and Xiong (2021) pointed out that after analyzing the evaluation indicators of four national universities in Japan, students’ learning behavior and emotional investment should be included in the evaluation index system, and more students’ “learning” should be included in the evaluation field. Tsou (2020) proposed to use AHP to integrate student evaluation, expert evaluation, and regular teaching assessment into the teaching evaluation system to form a new method of “same platform evaluation.” Ching (2019) believes that in order to develop relevant and constructive set indicators, the participation of important stakeholders, such as school managers, teachers and students, is essential, and more importantly, the service attributes that students want (power, rich experience and experience) should be taken into account.

### Contents of the teaching evaluation indicators of college students

In the specific content design of college students’ teaching evaluation indicators, Chinese scholars generally agree with the setting mode of secondary indicators. Yan and Wei (2016) believes that the setting of student evaluation indicators should follow the teaching principles of constructivism theory, highlight the core concept of teacher led and student-centered, design secondary indicators covering six aspects: teaching methods, teaching content, teaching attitude, teacher ethics and style, learning elements, learning effects, and set open questions for students to express their opinions and suggestions. Zhang et al. (2017) started with five first-level indicators of teaching attitude, teaching implementation, Teaching means and methods, teaching ability and level, and teaching effect based on literature research and teaching evaluation experience in colleges and universities. There are 33 secondary evaluation indicators based on learning theory and closely related to teaching quality, covering all aspects of the teaching process. Wu et al. (2015), from the perspective of systems theory, combined teaching evaluation theory, Chinese and other teaching evaluation cases and empirical research results, and designed a comprehensive index covering teaching enthusiasm, teaching organization, learning value, and teacher-student relationship, teaching content, teaching interaction, homework and assessment of 7 single indicators of evaluation index system. It is also worth mentioning that Zhang et al. (2019) optimized the student evaluation index system based on the new era’s requirements for higher education teaching quality, and constructed an index system of three levels: general education indicators, subject sharing indicators and school specific indicators, and added the relevant contents of “moral education” and “ideological politics” to the general education indicators.

Universities in some countries such as the United Kingdom, the United States, and Australia have set up special teaching evaluation and development institutions, whose members are composed of experts from different disciplines, and experts collectively discuss and formulate standardized student teaching evaluation scales. The evaluation indicators of the scale mainly refer to D. L stufflebeam’s CIPP evaluation model (Zhou, 2012). The CIPP model advocates helping managers (the makers of the indicator system) to systematically obtain and use evaluation feedback information in order to meet their needs or to utilize information resources as much as possible. Most of the colleges and universities in Western countries refer to this model, starting from the traditional student evaluation index, and divide it into three dimensions: background condition, process, and result to design evaluation index to systematically evaluate teacher teaching (Kellaghan and Stufflebeam, 2003; Zhao, 2010). Another SEEQ model has also been praised by foreign universities. The SEEQ evaluation index is composed of four parts: core index, characteristics of students and courses, additional index (supplementary questions) and open evaluation. Among them, the core index requires students to evaluate nine parts of teachers’ teaching. These nine parts include Academic, emotional, organizational, collaboration, personal communication, curriculum development, assessment, homework, and overall impression of teachers (Richardson, 2005; Marsh, 2007; Schellhase, 2010). Chinese scholars Jiang and Lu (2019) also found in their research on the students’ teaching evaluation system in ten first-class foreign universities that Stanford, MIT, Cornell and other colleges and universities evaluate students’ overall experience of the course and achieve the learning goals of the course. The evaluation of the situation and the evaluation of knowledge acquisition and skill development are included in the student evaluation index.

## Research on the problems and countermeasures in the practical application of student’s evaluation of teaching in higher education

Since the student evaluation system has been widely used in major colleges and universities in the world, it has not only achieved certain results, but also exposed many problems in practical application. The academic circles have abundant research results on the problems and improvement strategies in the practice of college students’ teaching evaluation. Although the opinions of various scholars are occasionally lacking, they are generally similar. This article summarizes the main points of view.

### Problems existing in the practice of teaching evaluation by college students

First, the function of teaching evaluation is alienated. There is a game between teaching managers, teachers and students in the existing student teaching evaluation system, that is, managers focus more on teachers’ “teaching” rather than students’ “learning.” Exaggerating the degree of teaching evaluation’s response to teachers’ teaching level weakens its function of teaching improvement (Becker and William, 2000; Jiang et al., 2018; Liang et al., 2020). Second, the evaluation index system is unscientific. According to the existing research, the unscientific aspects of teaching evaluation indicators are mainly reflected in the neglect of the subject status of students in teaching evaluation, the failure to distinguish the evaluation indicators of different professional courses, the too many invalid indicators and the complicated content, and the lack of theoretical guidance for the construction of the indicator system, etc (Ching, 2019; Sun, 2021). Constantinou and Wijnen-Meijer (2022) also pointed out that students, peers, curriculum managers and self-evaluation should be included in teaching evaluation (Chan, 2019). Third, the use of teaching evaluation results is unreasonable. In many colleges and universities, students’ teaching evaluation is a mere formality, only using quantitative scores to evaluate teachers’ performance, ignoring the value of qualitative teaching evaluation data; at the same time, the processing of teaching evaluation data is too simplistic, and a reasonable result feedback mechanism has not been formed (Chan, 2019). Fourth, the management system is imperfect. Restricted by subjective and objective conditions, at present, the management of students’ teaching evaluation in Colleges and universities at home and abroad is relatively extensive (Li et al., 2019), most of which are implemented by educational administration departments or entrusted to third-party evaluation institutions for operation, and few of them set up special organizations or establish clear rules and regulations to standardize the implementation of teaching evaluation.

### Improvement strategies for student’s evaluation of teaching in higher education

In view of the problems existing in the actual operation of College Students’ teaching evaluation, scholars at home and abroad have given suggestions for improvement from different angles. Sun and Sun (2020) believe that the failure of students’ teaching evaluation is caused by various games in teaching evaluation, and the fundamental solution is to change the function from the role of personnel management and summative evaluation of teachers. Tools, transforming into links and means of the ongoing process of diagnosing and developing teacher teaching. Long and Wang (2019) pointed out in their research that the use of the student teaching evaluation system should clarify the value, clarify the standards, and set the rules, and conduct a comprehensive evaluation from the aspects of clarifying the purpose of evaluation, optimizing the evaluation indicators, enriching the evaluation forms, and rationally using the results. It is comprehensively constructed to realize the teaching academic value of students’ evaluation of teaching. Xu (2017) believes that timely self-improvement is an important part of the new student teaching evaluation system, so the student teaching evaluation process should be optimized based on the principle of continuous improvement, and a problem tracking and monitoring guarantee mechanism should be established. Through his research, Svinicki (2010) showed that open evaluation plays an important role in students’ teaching evaluation. The limitation of pure quantitative evaluation on students’ expression should be reduced as much as possible and more open possibilities should be provided in terms of teaching evaluation indicators. Marsh and Herbert (1987) put the perspective on the feedback of teaching evaluation results, and believed that the influence and effectiveness of students’ teaching evaluation results can be expanded through three feedback methods: summary of students’ teaching evaluation results, summary materials for each teacher, and teaching expert advice given in combination with students’ teaching evaluation results. Hassanein et al. (2012) concluded from a SET study conducted in nursing schools that improving the teaching evaluation process must take into account the diversity of student characteristics, student evaluation goals, teaching methods, and institutional context.

## Comments on existing research

### Research perspective

The original intention of the student evaluation system is to let students, as the main body of teaching, evaluate teachers’ teaching behavior. However, with the popularization and development of the system in colleges and universities around the world, the conflicts of interest among teaching managers, teachers and students in teaching evaluation are gradually revealed. In this game, managers put the focus of students’ teaching evaluation on teachers, and take teaching evaluation as a simple and effective tool to measure teachers’ performance. In fact, students’ expression of teachers’ teaching is limited and controllable. In the current research results, scholars at home and abroad have a more profound understanding of the absence of students’ evaluation of teaching, generally shifting the research perspective to the concept of “student-centered” evaluation of teaching, and considering “the actual needs of students” and “promoting the all-round development of students” in the research of various parts such as the function, content and results of evaluation of teaching. However, the current research perspective is lack of comprehensiveness, and the seemingly reasonable transformation cannot resolve the contradiction between the three in the student evaluation system. Overemphasizing the student standard will magnify the deviation of the system in the evaluation of teachers’ teaching quality, and increase the cost and burden of teaching managers.

### Research contents

The academic research on college students’ teaching evaluation mainly focuses on reliability, validity and its influencing factors, evaluation indicators, deficiencies in practical application and improvement strategies, among which the research results on the effectiveness of teaching evaluation are the most abundant. With the wide application of the student teaching evaluation system, the research scope of validity has expanded from the initial analysis of rationality to the research on the reliability of students’ teaching evaluation results and the final validity of students’ teaching evaluation. In terms of influencing factors, although researchers have formed a relatively unified view on its main categories, due to the large number of subtle factors and different research perspectives, the influence results of specific factors are also different, making the research results of this part complex and full of controversy. In terms of evaluation indicators, the research on the theory of index construction has been relatively complete, and the combination of qualitative and quantitative evaluation can generally be used, which reflects the academic quality of index construction. However, there are still different strengths and weaknesses in the design of specific evaluation indicators, and there is a lack of a comprehensive, systematic and authoritative index design framework, so it is difficult to form a unified opinion and promote its application. In terms of problems and countermeasures in practice, scholars at home and abroad have relatively unanimous opinions on the problems existing in the current teaching evaluation process of college students, and have carried out a relatively comprehensive analysis. However, the countermeasures proposed for the problem are too vague and unconvincing, and it is necessary to further verify and concretize them in the application process to obtain more effective improvement suggestions.

### Research methods

At present, scholars’ research on students’ teaching evaluation system is no longer limited to literature, but more to empirical investigation and statistical analysis. In recent years, in terms of the reliability and validity of students’ teaching evaluation and its influencing factors, more and more researchers have used statistical methods such as independence test, multiple regression analysis, ordered regression model (OLM) to analyze the data of students’ teaching evaluation. The correct and reasonable use of appropriate data processing methods has significantly improved the scientificity of the research on the effectiveness of students’ teaching evaluation. In order to build a scientific and reasonable evaluation index, researchers prefer to use questionnaires, interviews, random sampling and other methods to conduct empirical research on the subjects and cases of teaching evaluation in colleges and universities. However, a good empirical study is extremely difficult to operate, which requires a large sample size and will also cost more time, human resources and other resources. Therefore, the evidence of empirical research in the current results is still relatively shallow, and we can try to combine it with big data and artificial intelligence algorithms to supplement it with diversified research methods.

### Research trends

According to the current research trend in academia, first of all, researchers will continue to explore the influencing factors of students’ teaching evaluation. The influencing factors of college students’ teaching evaluation are extremely complex, but it is extremely important to overcome the negative effects of interfering factors and improve the limitation of students’ teaching evaluation. Therefore, the research on this issue in academic circles will continue to deepen. Secondly, the rapid development of the Internet has innovated the form of students’ teaching evaluation, and students’ online teaching evaluation has become the current mainstream model. While online teaching evaluation brings convenience to the students’ teaching evaluation system, new problems such as the weakening of the realism of the scene and the difficulty of supervising the process have also appeared. In addition, how to solve the shortcomings of the online teaching evaluation system, such as strong subjectivity of students, low teacher participation, and imperfect application of result feedback, is also becoming a problem worth exploring for researchers. Finally, the integrated development of multi-disciplinary and multi-angle will be the key direction of future research on the teaching evaluation system of college students. Scholars have found in their research that the content involved in the student evaluation system is far beyond the field of education, and its scope also covers psychology, sociology, statistics, economics and many other disciplines. Therefore, some researchers have integrated and analyzed student evaluation of teaching with other disciplines. It is foreseeable that in future research, scholars will view the improvement and development of the student evaluation system from a multidisciplinary perspective.

## Conclusion and recommendations

In this article, we have provided a more in-depth analysis of the theories and methods of student’s evaluation of teaching in higher education in China and abroad, and point out the desirable experiences and shortcomings of them. Firstly, in terms of the reliability and validity of student’s evaluation of teaching, the current research has made great progress in terms of validation methods, and some scholars have been able to use appropriate data analysis models to improve the persuasiveness of the results. Secondly, the research on the theory and structure of the evaluation indicators for university students generally revolves around the “student-centered theory” and “secondary indicator structure,” the rationality of which has been confirmed; however, the academic circle has not yet formed a unified opinion or standard on the selection of the specific content of the evaluation indicators. However, there is no unified opinion or standard on the selection of the specific content of evaluation indicators, and fewer scholars have paid attention to the differentiation of the indicators for different courses. Finally, regarding the problems and countermeasures in the application of student’s evaluation of teaching in higher education, current research has analyzed the process of student’s evaluation of teaching from the aspects of purpose, indicator system, application of results and process management in an all-round way and found the shortcomings, however, the scholars’ expressions of improvement measures are still abstract and lack of pertinence, making it difficult to carry out concrete operations. In view of the above analysis, this article improves the research on student’s evaluation in Western higher education from a Chinese perspective, and at the same time provides a reference for the construction of an indicator system for student evaluation in Chinese universities, taking into account the actual situation of Chinese universities. The future research will be based on the successful experience of student evaluation in universities, improve the problems, explore the influence mechanism of different factors on student evaluation in universities, optimize the indicator system and management system of student evaluation, especially the indicator system of student evaluation in ideological and political theory courses in Chinese higher education will be constructed in accordance with the characteristics of Chinese ideological and political theory courses, so as to improve the teaching quality of ideological and political theory courses in Chinese higher education. We will make our contribution to improving the teaching quality of ideological and political theory courses in Chinese higher education.

## Author contributions

SY: conceptualization, resources, writing-review and editing, and supervision. SY, LZ, and PX: methodology. LZ and PX: formal analysis. LZ and YC: investigation. SY and YC: project administration. All authors contributed to the article and approved the submitted version.

## Funding

This research was funded by National Innovation and Entrepreneurship Training Program for Undergraduate (No. 202210316091Y).

## Conflict of interest

The authors declare that the research was conducted in the absence of any commercial or financial relationships that could be construed as a potential conflict of interest.

## Publisher’s note

All claims expressed in this article are solely those of the authors and do not necessarily represent those of their affiliated organizations, or those of the publisher, the editors and the reviewers. Any product that may be evaluated in this article, or claim that may be made by its manufacturer, is not guaranteed or endorsed by the publisher.
